# Biphasic synovial sarcoma of the hypopharynx: Case report and literature review

**DOI:** 10.1016/j.ijscr.2022.106784

**Published:** 2022-01-24

**Authors:** Felipe Girón, Lina Rodriguez, Carlos Eduardo Rey Chaves, Marcela Estrada, Fernando Gutierrez, Andrés Álvarez

**Affiliations:** a Faculty of Medicine, Universidad del Rosario, Bogotá, Colombia; b Faculty of Medicine, Universidad de los Andes, Bogotá, Colombia; c Hospital Universitario Mayor Méderi, Colombia

**Keywords:** Synovial sarcoma, Head & neck, Surgery, Case report

## Abstract

**Introduction:**

Synovial Sarcoma is a rare malignancy that accounts between 8 and 10% of soft tissue neoplasms, with the highest presentation rate in extremities, an extremely uncommon condition in head and neck.

**Clinical findings:**

We present a case of an 18-year-old male with synovial sarcoma situated at hypopharynx who underwent surgical resection and postoperative radiotherapy.

**Conclusion:**

Synovial Sarcoma represents a rare head and neck malignancy with challenging diagnostic approach due to its frequency and nonspecific clinical manifestations. Surgical treatment must assure good free margins. Adjuvant radiotherapy has a positive impact in local recurrence and survival.

## Highlights

• Sinovial Sarcoma it's a rare and challenging entity, early diagnosis is related with better outcomes.
• On time adjuvant therapy needs to be done in order to improve oncologic outcomes.
• Multidisciplinary approach it's related to good postoperative outcomes and extended overall survival.

## 1 Introduction

Synovial Sarcoma is a rare malignancy that accounts between 8 and 10% of soft tissue neoplasms, with a higher presentation rate in extremities, and extremely uncommon in head and neck, and in this location the hypopharynx it's the most common site of occurrence, other locations include: prevertebral and parapharyngeal areas, laryngeal, and nasopharynx [1], [2]. Diagnosis is challenging due to its low frequency and nonspecific clinical manifestations, however clinical manifestations appear when the size it's enough to compromise adjacent structures in cases of head & neck, these features differ from the ones of the extremities, and diagnosis could be delayed an average of 20 months [2]. Also, head & neck synovial sarcoma shows a better prognosis, due to a lower rate of growth through the years, and a less probability to infiltrate lymph nodes [2]. Literature shows a survival rate between 40 and 60% at 5 years of follow up, that could be significantly lesser than the patients with extremities malignancies (35–45%) [2]. After the first description no more than 90 cases are reported in the literature. We present a case of an 18-year-old male with synovial sarcoma situated at hypopharynx who underwent surgical resection and postoperative radiotherapy. Surgical treatment must assure good free margins and adjuvant radiotherapy has shown to have a positive impact in local recurrence and survival. Work has been reported in accordance to SCARE guidelines [3].

## 2 Case presentation

Previous informed consent filled, following SCARE guidelines [3]. An 18-year-old male presented with odynophagia, dysphagia, and left ear pain. Initial physical examination was unremarkable. A swallowing disorder was considered, and a video-fluoroscopic study was obtained, showed a hypopharynx mass with multiple aspirated episodes. Neck CT scan with contrast showed polypoid mass in hypopharynx originating in the left wall from the free margin of the epiglottis to the aryepiglottic folds. Mass measured 62 × 33 × 25 mm and caused partial occlusion of the laryngeal vestibule.

A month later, the patient presented to the emergency room in a different hospital with dyspnea and worsening of dysphagia leading to inability to swallow solids or liquids, reported weight loss of 3 kg in the last month and frequent cough. Palpable IB lymph nodes were found during physical exam. Patient was admitted.

**Fig. 1:**
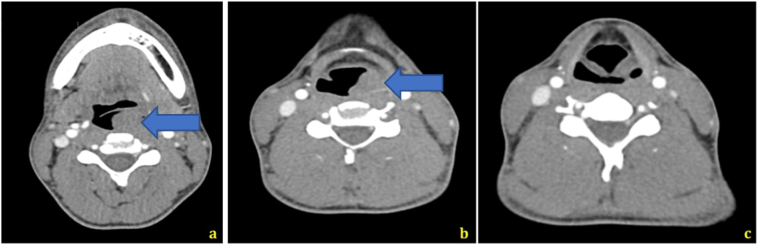
Tomographic findings. (a: >60% occlusion of the oropharynx b and c: Mass in the pharynx's posterolateral wall and involving retro cricoid region c:) Arrows pointing mass.

A new neck Computed Tomography (CT) scan was obtained due to lack of report from the previous and showed a 91 × 27 × 50 mm mass located in pharynx's posterolateral wall and involving retro cricoid region, extending from the pyriform sinus to the oropharynx and causing an occlusion greater than 60% (Fig. 1). After consulting with an otorhinolaryngologist the decision to do a flexible endoscopy was made. Study revealed a left pharyngeal mass in the oropharynx and hypopharynx, dependent on left glossoepiglottic and aryepiglottic folds with normal vocal cords mobility, a biopsy was obtained. Histological analysis of the mass sample revealed a biphasic synovial sarcoma. Immunohistochemical staining was positive for CK7, BCL-2, TLE-1 (weak positive for mesenquimal component), P63 and synaptophysin focal positive for glandular component, and negative for CD34, SALL4, desmin and STAT6.

The patient was referred to our surgical oncology department where the case was discussed by specialists in oncology and head and neck surgery. Due to the frequent metastases and lymph node involvement of this pathology a Positron Emission Tomography (PET)/CT was obtained. The scan showed a malign infiltrative mass compromising the left oropharynx and hypopharynx. Additionally, laterocervical lymph nodes (in regions IB right, IIA bilateral predominantly right and III left) with low FDG affinity were found. Bone scintigraphy was performed to rule out metastases with negative results. As well, PET Scan do not show any hypermetabolic capitation in distance places, ruling out metastatic disease.

**Fig. 2:**
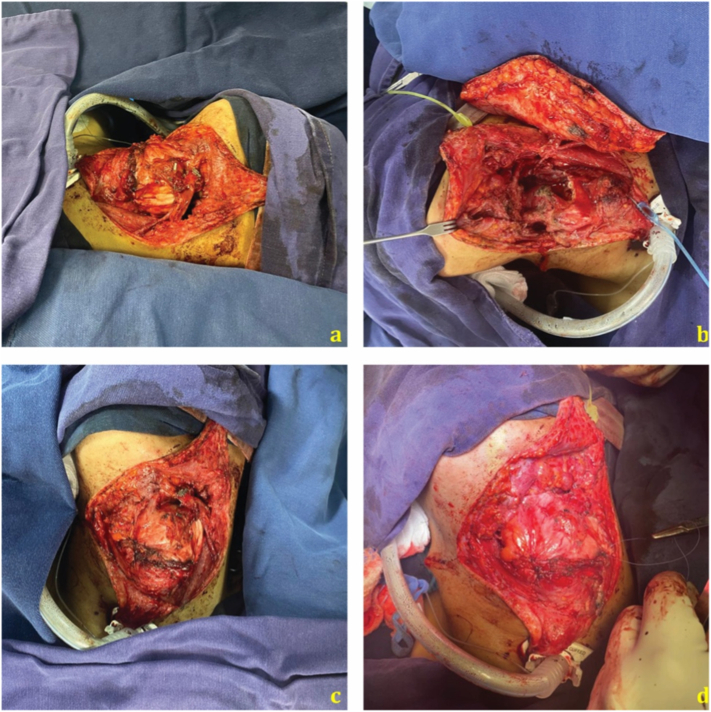
Intraoperative findings. (a: Neck dissection b: Pharynx exposure c: Mass dissection d: Unilateral lymphadenectomy).

**Fig. 3:**
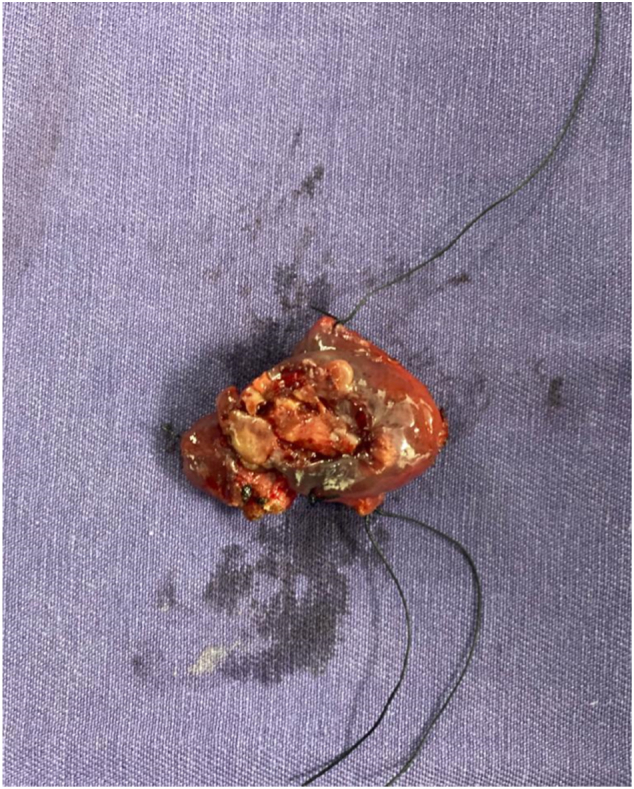
Surgical piece.

After the multidisciplinary discussion and analysis of the case the patient underwent an excision of the mass through a pharyngectomy with unilateral lymphadenectomy by an experienced head & neck surgeon (Fig. 2). During surgery the tumor was dissected from the posterolateral pharynx wall and the left pyriform sinus without compromising the epiglottis (Fig. 3). Enlarged lymph nodes in groups II, III and V were identified and resected, reconstruction was made using an Antero-lateral Thigh flap. Gastrostomy was performed to protect the reconstruction made. Cytogenetic study revealed 95% of nuclei rearrangement in 18q 11.2.

Following surgery, the patient did not require management in the intensive care unit and had an uneventful immediate post-operative recovery, without nerve deficit. The patient was discharged home. Received Intensity Modulated Radiotherapy (IMRT) with a daily dose of 100 cGy in 25 fractions for a total dose of 6000 cGy.

At 6 months post hypopharynx tumor resection the patient continues with uneventful follow up, no signs of local relapses. He has resumed his regular life.

## 3 Discussion

Synovial Sarcoma (SS) is an uncommon soft tissue malignancy accounting for 8–10% of soft tissue neoplasms [1], [2]. SS is named due to its histological resemblance to synovium found in articular tissues especially on the knee, however there is no biological relation between SS and synovial tissue [1], [2]. Its cellular origin is still unclear, but it is derived from pluripotent mesenchymal cells [4]. SS is more frequent in the younger population (15–29 years old) with the highest incidence on extremities [2], [4], [5], with a slightly higher relation towards men (3:2) and higher survival in women [6]. There are no other risk factors known to be associated with SS appearance. Head and neck synovial sarcomas have been reported in paravertebral, superior aerodigestive, mandibular, parapharyngeal, retropharyngeal and hypopharyngeal tissue, being the last one the most frequent location. It was first described by Paul Jernstrom in 1954 [1], [5].

Head and neck synovial sarcomas usually present as a non-painful mass with slow progressive growth hence its vague clinical presentation. Consequently, symptoms will depend on mass effect over adjacent structures and may range from mild throat discomfort, hoarseness to dysphagia and dyspnea [2], [5]. SS are multinodular masses that may vary diameter from 1 to 15 cm [5], [7], [8], they can be classified into monophasic or biphasic. Monophasic synovial sarcomas (MSS) can be epithelial, poor differentiated, calcified, and mixed due to their histological characteristics [4], [5]. MSS is more frequent, characterized by the presence of only one cellular type (Fusiform or epithelial) that can be sometimes interpreted as fibrosarcoma, hemangiopericytoma or neurofibrosarcoma. On the other hand, a biphasic synovial sarcoma (BSS) is characterized by presence of fusiform and epithelial cells with an important macroscopical hemorrhagic and necrotic component [4], [5], [10], [11]. Tumor size is associated with histological characteristics being MSSs smaller than 5 cm and BSSs bigger than 5 cm, both are frequently associated with bone metastases [2], [5], [6], [7].

Preoperative diagnosis is challenging due to its rare occurrence and nonspecific clinical presentation, being images the most important tools in its identification and diagnosis [6], [7]. Magnetic Nuclear Resonance (MRI) is preferred for initial assessment and stadification due to its capability of showing muscular, bone and neurovascular invasion [9]. Conversely, Computed Tomography (CT) can also be used, usually identifying an hypo intense image with well-defined borders. CT is useful for evaluation of airway compromise and compression. Lymph node invasion in head and neck SS is seen in CT in 12.5% of cases [8], [9]. There is no correlation between imaging and histological findings [8], [9]. On the other hand, immunohistochemistry of synovial sarcomas is complex and unique, it includes mesenchymal markers expression such as vimentin and epithelial markers as cytokeratins 19 and 7 and epithelial membrane antigen that are presented in 90% of all cases [4], [5], [10], [11], [12]. It is important to consider that fusiform cell marker expression can be focal, therefore examination and immunohistochemistry study of different sections is recommended [10], [11], [12]. S100 protein and CD 99 are also recommended due to its expression in fusiform and epithelial cells even though their presence is not diagnostic [4], [5], [13].

Furthermore, translocation t(X;18)(p11.2;q11.2) between chromosomes 18 (SYT gene) and X (SSX gene) is associated with synovial sarcomas [4], [5], [6], [10]. The resultant copies SYT-SSX1 and SYT-SSX2 will encode an aberrant nuclear transcription factor that alters chromatin remodeling, which in turn modifies gene expression patterns. SYT-SSX-2 translocation is associated with MSS, and SYT-SSX1 translocation with BSS [4], [5], [6], [10]. Biphasic synovial sarcomas are related to higher proliferation rates and therefore worse prognosis [11], [13], [14].

Overall, surgery is the election treatment and performing a wide local resection ensuring 2–5 cm free margins is fundamental [11]. Recurrence rate of head and neck SS after surgical procedure may vary between 21 and 56% depending on margins and histological characteristics [5], [14], [15]. Prophylactic lymph node dissection is not recommended due to infrequent lymphatic dissemination, however when positive lymph nodes are documented preoperatively, dissection must be performed [4], [5], [12], [15]. Adjuvant radiotherapy must be considered to reduce recurrence rate and increase survival [16]. Neoadjuvant chemotherapy must be considered in patients with tumor size greater than 5 cm or clinical or imagenological evidence of local extension to vital structures even though its use is controversial [10], [11], [12], [15].

Lastly, synovial sarcomas have poor prognosis with an average survival rate at 5 years of 25–62% and a 10-year survival rate of 11–30% [4], [5], [15]. Half of the cases present late metastases with lung, bone and lymph nodes being the most common. Patients older than 25 years old, tumors larger than 5 cm and poor tumoral differentiation have an 18% chance of living without recurrence [11], [14], [15]. Poor prognostic factors include delay in diagnosis from the start of symptoms, age of presentation older than 25 years old, tumors larger than 5 cm, thoracic or abdominal involvement, poor tumoral differentiation, mitotic index >10/10 CGA, extense tumoral necrosis in histology, high grade of cell atypia and aneuploidy, Ki-67 >10%, bone metastases and lack of extensive margins during resection [4], [6], [11], [12], [15]. Patients with head and neck SS have a local recurrence in 45% of cases and 33% develop distant metastases [4], [7]. This case report increased the evidence of patients with head and neck synovial tumors, that actually, are less than 100 in the worldwide literature, and also shows the importance of multidisciplinary approach of these complex cases.

## 4 Conclusion

Synovial Sarcoma represents a rare head and neck malignancy with a challenging diagnostic approach due to its frequency and nonspecific clinical manifestations. Surgical treatment must assure good free margins. Adjuvant radiotherapy has a positive impact in local recurrence and survival.

## Provenance and peer review

Not commissioned, externally peer-reviewed.

## Funding information

This research did not receive any specific grant from funding agencies in the public, commercial, or not-for-profit sectors.

## Ethical approval

Ethical approval of institutional committee was made previous publication.

## Consent

Written informed consent was obtained from the patient for publi-cation of this case report and accompanying images. A copy of the written consent is available for review by the Editor-in-Chief of this journal on request.

## CRediT authorship contribution statement

**Felipe Giron, MD, MSc**: Make substantial contributions to concep-tion and design, acquisition of data,analysis and interpretation of data.

**Carlos Rey, MD**: Participate in drafting the article and revising it critically for important intellectual content.

**Lina Marcela Rodriguez, MD**: Participate in drafting the article and revising it critically for important intellectual content.

**Fernando Gutierrez, MD**: Participate in drafting the article and revising it critically for important intellectual content.

**Andrés Álvarez, MD**: Participate in drafting the article and revising critically for important intellectual content. Give final approval of the version to be submitted and any revised version.

**Marcela Estrada MD**: Participate in drafting the article and revising critically for important intellectual content.

## Research registration number

None.

## Guarantor

Felipe Giron.

## Declaration of competing interest

Authors do not declare any conflict of interest.
